# Advances of MnO_2_ nanomaterials as novel agonists for the development of cGAS-STING-mediated therapeutics

**DOI:** 10.3389/fimmu.2023.1156239

**Published:** 2023-04-19

**Authors:** Tangxin Zhang, Chunmiao Hu, Wenting Zhang, Yongdui Ruan, Yuhe Ma, Dongsheng Chen, Yuhe Huang, Shuhao Fan, Wensen Lin, Yifan Huang, Kangsheng Liao, Hongemi Lu, Jun-Fa Xu, Jiang Pi, Xinrong Guo

**Affiliations:** ^1^ Guangdong Provincial Key Laboratory of Medical Molecular Diagnostics, The First Dongguan Affiliated Hospital, Guangdong Medical University, Dongguan, China; ^2^ Dongguan Key Laboratory of Environmental Medicine, School of Public Health, Guangdong Medical University, Dongguan, China; ^3^ Institute of Laboratory Medicine, School of Medical Technology, Guangdong Medical University, Dongguan, China

**Keywords:** MnO_2_ nanomaterials, manganese ions, cGAS agonist, cGAS-STING pathway, novel therapeutics

## Abstract

As an essential micronutrient, manganese plays an important role in the physiological process and immune process. In recent decades, cGAS-STING pathway, which can congenitally recognize exogenous and endogenous DNA for activation, has been widely reported to play critical roles in the innate immunity against some important diseases, such as infections and tumor. Manganese ion (Mn^2+^) has been recently proved to specifically bind with cGAS and activate cGAS-STING pathway as a potential cGAS agonist, however, is significantly restricted by the low stability of Mn^2+^ for further medical application. As one of the most stable forms of manganese, manganese dioxide (MnO_2_) nanomaterials have been reported to show multiple promising functions, such as drug delivery, anti-tumor and anti-infection activities. More importantly, MnO_2_ nanomaterials are also found to be a potential candidate as cGAS agonist by transforming into Mn^2+^, which indicates their potential for cGAS-STING regulations in different diseased conditions. In this review, we introduced the methods for the preparation of MnO_2_ nanomaterials as well as their biological activities. Moreover, we emphatically introduced the cGAS-STING pathway and discussed the detailed mechanisms of MnO_2_ nanomaterials for cGAS activation by converting into Mn^2+^. And we also discussed the application of MnO_2_ nanomaterials for disease treatment by regulating cGAS-STING pathway, which might benefit the future development of novel cGAS-STING targeted treatments based on MnO_2_ nanoplatforms.

## 1 Introduction

Host cells initiate innate immune responses by recognizing conserved pathogen structures through pathogen-associated molecular patterns (PAMPs) and host damage-associated molecular patterns (DAMPs) through diverse pattern recognition receptors (PRRs), both of which can identify abnormal DNA (1, 2). Cyclic GMP-AMP synthase (cGAS, cGAMP synthase) is a cytosolic DNA sensor belonging to the nucleotidyltransferase family. When binds to DNA, cGAS undergoes a conformational change to an active state and generates the second messenger circulating GMP-AMP (cGAMP) to activate a type-I interferon response by binding to STING, which coordinates immune defense mechanisms (3, 4). The cGAS-STING axis is activated not only by non-self DNA, but by extracellular,mitochondrial and nuclear DNA capable of entering the cytoplasm. Elevated levels of cytoplasmic DNA due to inflammation, autoimmunity, tumors, bacteria and viruses would lead to constitutive and systemic activation of cGAS-STING and promote the body’s immune defense. Increasing pieces of evidence suggest that cGAS-STING pathway is important in mediating cellular immune sensing, which therefore can be used as novel targets for the vaccine and drug development (5, 6).

In recent years, manganese has been widely concerned as an agonist of the cGAS-STING pathway. Manganese is the twelfth most abundant element on the planet, and it is an indispensable trace element in the normal human body. As the catalytic center and the structural core of various enzymes, manganese is involved in a large number of biological processes, including oxidative phosphorylation, glycosylation and signal transduction (7, 8). Due to these critical functions, manganese has been proved to play critical roles in some important diseases. For example, manganese ions (Mn^2+^) can regulate the complicated tumor microenvironment, including hypoxia regulation, glutathione depletion, glucose consumption and immunosuppressive tumor microenvironment regulation, which therefore introduce manganese for anti-tumor strategy development (9, 10). Combined with glucose oxidase, Mn^2+^ can also indirectly convert glucose into ROS to induce oxidative damage of tumor tissue, which might result in better anti-tumor activity of current chemotherapies (11). Moreover, manganese is also capable of regulating the immune system dependent on different mechanisms. For example, Mn^2+^ can effectively promote DC maturation and antigen presentation of DC and macrophages to augment CD8+ T cell and NK cell activation (12).

Moreover, manganese can activate the host immune system by regulating the cGAS-STING pathway (13, 14). It has been widely known that Mn^2+^ can enhance the sensitivity of cGAS to double-stranded DNA (dsDNA) and its enzymatic activity by binding with cGAS to enhance cGAMP-STING binding affinity (15, 16). Mn^2+^ directly activates cGAS without DNA and triggers a distinct catalytic synthesis of 2’3’-cGAMP,so Mn^2+^ can induce cells to produce type I IFNs without any infection (16, 17). Mn^2+^ can catalyze H_2_O_2_ into • OH, a kind of highly active reactive oxygen species (ROS) that is helpful to activate cGAS-STING signalings (18). Mn^2+^ also shows STING-independent immune activating potential by inducing phosphorylation of TBK1 and p65, which is further augmented and translated to IRF3 phosphorylation in the presence of STING agonists, resulting in amplification of the STING signalling cascade (19). Based on the above ability, manganese seems to bridge innate and adaptive immunity by promoting DC maturation and antigen presentation (17), which therefore shows their potential for anti-tumor and anti-infection treatments (8, 16, 20, 21).

The rapid development of nanotechnology in recent decades allow the more and more clinical application of functional nanomaterials (22, 23). Due to the complicated valence of manganese, there are different kinds of manganese nanomaterials, and MnO_2_ nanomaterials (MnO_2_ NPs) are one of the most stable and functional nanomaterials among them. MnO_2_ NPs have shown a wide range of applications in biomedicine, such as biological imaging therapy, drug delivery and immune regulation. MnO_2_ NPs can be used as an ideal biodegradable agent for specific drug delivery, while producing Mn^2+^ (24, 25). This can significantly promote the Fenton-like effect to amplify the generation of cytotoxic hydroxyl radicals (• OH) and achieve significant GSH depletion enhanced chemokinetic therapy (CDT) (25). Additionally, MnO_2_ is one of the best alternative contrast agents for T1-weighted magnetic resonance imaging (MRI) besides gadolinium-based contrast agents (24). Enzymatically synthesised MnO_2_ NPs (Bio-MnO_2_ NPs) also possess photothermal therapy (PTT) functions because of their extraordinary photothermal conversion efficiency (44%) (26). Additionally, MnO_2_ NPs, as a kind of TME-responsive drug carrier, can be rapidly decomposed in acidic and reduced environment to show on-demand drug release at the tumor sites, which highlighted the critical roles of MnO_2_ NPs in cancer therapy and theranostics.

Mn^2+^ produced by MnO_2_ NP in cells can regulate the cGAS-STING signal pathway and promote the pathway to play an important physiological function. This indicates the potential ability of MnO_2_ NP to target the cGAS-STING signal pathway for disease prevention and treatment.In this review, we summarized the ability and mechanism of MnO_2_ NPs to regulate cGAS-STING signaling events. Moreover, we also introduced the current progress of MnO_2_ NPs for potential disease treatment by regulating GAS-STING pathway, which might provide more possibilities to develop novel therapies in different disease conditions.

## 2 Biological characteristics of cGAS-STING pathway

### 2.1 Structure and function of cGAS

cGAS is an unstructured, highly basic protein of approximately 160 amino acid amino-terminal (N-terminal) domain and a globular approximately 360 amino acid domain of 520 amino acid protein (4, 27, 28). The catalytic domain of cGAs consists of two structural lobes in which the active site is located (4, 29, 30). Lobe 1 includes the evolutionarily conserved NTase core β-Sheet and a conserved acidic residue involved in the Mg^2+^-dependent catalytic transfer of nucleoside phosphates to hydroxyl acceptors is also found in DNA polymerases. Lobe II completes the active site and increases the interaction for nucleoside triphosphate donor binding (4).

Free cGAS (not bound to dsDNA) is not catalytically active because it has no catalytic site of the suitable structure. When cGAS is activated by dsDNA, binding of dsDNA (length > 45 b) between two cGAS monomers creates a structured catalytic site that results in cGAS activation. The structure of cGAS activation is a dimer with the two DNA strands sandwiched between two cGAS protomers (29, 30). This allosteric process of cGAS dimer formation converts cGAS from an inactive to an active state. There are two binding sites “ a “ and “ b “ for each cGAS protomer in the dimer, with one DNA strand binding to site “ a “of one cGAS protomer and site “b” of the other corresponding cGAs protomer. cGAS covers approximately 16-18 bp of DNA, and this binding is independent of the sequence information bound (4, 29, 30).

As a DNA receptor, cGAS can recognize different types of DNA, which may be related to different subcellular structural localization (4). However, the cellular localization of cGAS is still unclear. cGAS may exist in the nucleus, cytoplasm and cell membrane, because highly disordered N-terminal fragments of cGAS may play a role in the attachment of cGAS plasma membrane (31). As a DNA receptor, cGAS can recognize exogenous DNA, including infection related DNA from viruses and viruses or bacteria entering the cytoplasm (32, 33), as well as self sourced DNA, including mitochondrial cytoplasmic DNA (34), DNA in cytoplasmic micronucleus (35) and chromatin in nucleus. Structurally, it includes long dsDNA molecules, single strand DNA (ssDNA) with local secondary structure, short synthetic DNA with G-rich single strand protrusions, and RNA DNA hybrids (but RNA DNA hybrids constitute sub optimal agonists) (4).

### 2.2 Signaling processes and functions of cGAS-STING pathway

**Figure 1:**
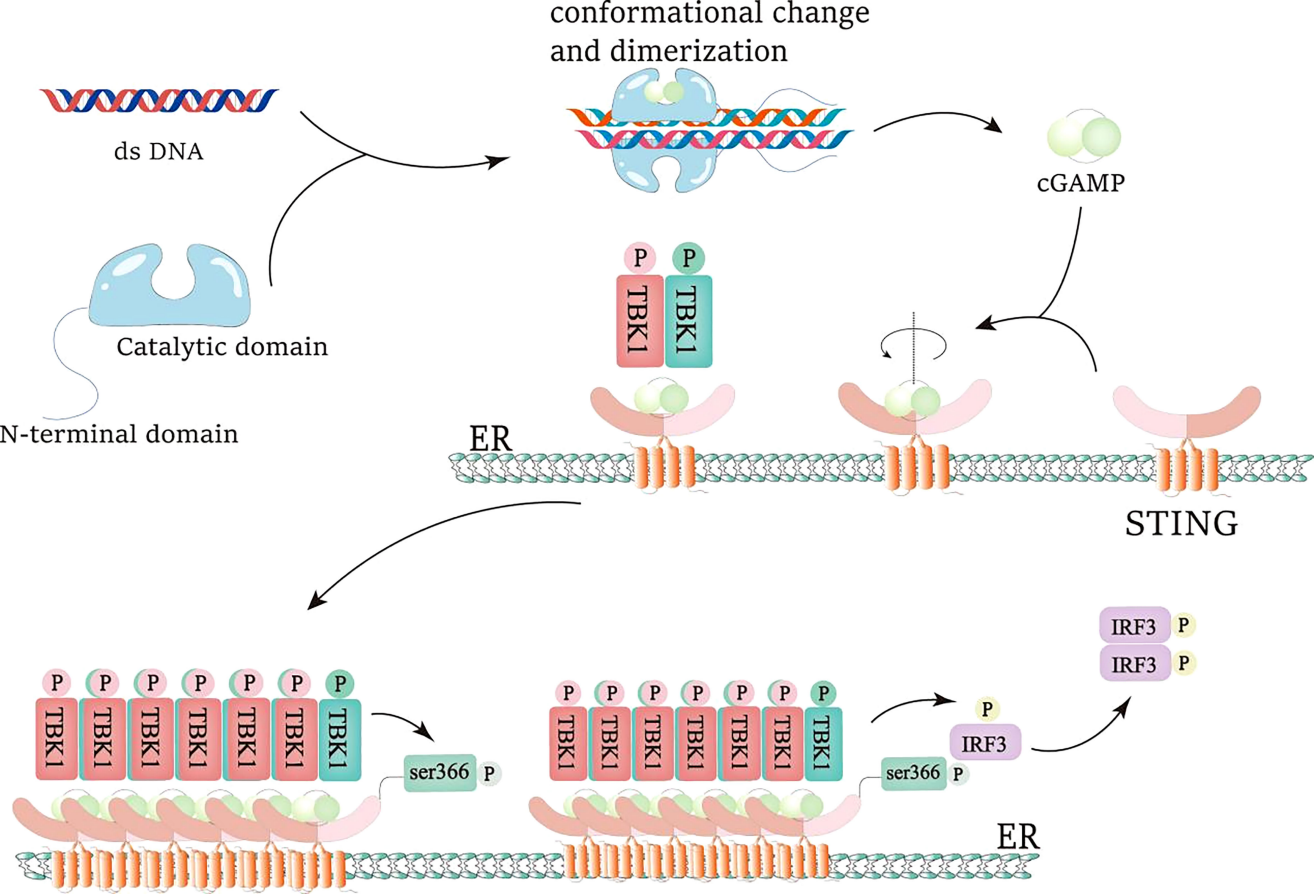
IRF3 activation of cGAS-STING pathway. dsDNA is recognized and combined by cGAS, resulting in the conformational change and dimerization of cGAS and the generation of cGAMP. When cGAMP is combined with the STING pocket structure, the STING dimer rotates 180°relative to the transmembrane helmets, which is beneficial to the oligomerization of the STING dimer and the promotion of the trans-phosphorylation of TBK1. Active TBK1 generates the docking site of IRF3 by phosphorylating the S366 site of STING. Then, active TBK1 phosphorylates IRF3, promotes IRF3 dimerization, and makes IRF3 obtain transcriptional activity.

The classical biological function of cGAS is an indispensable bridge between the recognition of exogenous pathogen dsDNA and immune defense. In summary as shown in **Figure 1**, the combination of cGAS and dsDNA induces the dimerization of cGAS and leads to conformational changes, promoting the oligomerization and activation of cGAS (36). The cGAS dimer combines with two molecules of dsDNA to initiate liquid-liquid phase separation and form a trapezoidal structure, which enables cGAS to be allosterically activated and opens the catalytic pocket to catalyze the cyclization of ATP and GTP to form cGAMP (28, 36). cGAMP is the first cyclic dinucleotide found in multicellular animals and acts as an endogenous second messenger to trigger downstream cascade signals (28).

The most important and typical downstream target of cGAS is STING signal pathway (4, 28, 37, 38). cGAMP combines with the small pocket of dimer STING to change the conformation of STING and promote the transportation of STING from endoplasmic reticulum to Golgi apparatus (4, 28). In this process, STING raised TANK binding kinase 1 (TBK1) to activate its autophosphorylation. Activated TBK1 can phosphorylate STING in turn, and enhance the interaction between STING and interferon regulatory factor 3 (IRF3), thereby further promoting TBK1’s phosphorylation of IRF3. Phosphorylated IRF3 dimerizes and enters the nucleus, thereby inducing transcription of type I interferon (IFN-1) and other cytokines (4, 28, 37). IFN-1 receptor is a heterodimer composed of IFNAR 1 and IFNAR 2 subunits (39). IFNs first bind a high-affinity receptor chain (IFNAR 2), and then recruit a low-affinity receptor chain (IFNAR 1) to produce a signal-active ternary complex. Receptor dimerization activates Tyk 2 and JAK 1 kinases that phosphorylate STAT 1 and STAT 2. The phosphorylated STAT 1 and STAT 2 heterodimers combine with IRF 9 to produce the transcription factor ISGF 3. ISGF 3 binds to ISREs and promotes the expression of a large number of ISGs. ISG can target different stages of virus replication and then resist virus infection. Unlike IFN-α, IFN-β can form a high-affinity complex with IFNAR 1 and conduct IFN-b signal transduction in an IFNAR 2 independent manner. In addition to their antiviral effects, IFN-I is also protective against several extracellular bacterial infections (40). For example, in Streptococcus, Escherichia coli, and Helicobacter infections, the increased expression of IFN-I is beneficial to anti-bacterial infection. At the same time, STING can also activate IκB kinase (IKK), and phosphorylate NF-κB Inhibitory Protein IκB αSer32 and Ser36, leading to their polyubiquitination and degradation, followed by NF-κB can enter the nucleus. The cGAS-STING-IKK signaling pathway, together with IRF3, induces classical NF-κB function, starts the transcription of immune stimulating gene, and passes the non classical NF-κB Pathway participate in other important activities (28, 37, 38, 41). cGAS recognizes abnormal exogenous and endogenous DNA, promotes activation of multiple immune stimulators, autophagy, remodeling of tumor environment, and production of inflammatory factors through cGAS-STING signal pathway, and plays an important role in inflammation, autoimmunity, anti-tumor, anti-bacterial, and viral infection (28, 32, 33, 38, 42–46).

cGAMP is a kind of cyclic dinucleotide (CDN) formed by the connection of GTP and ATP through phosphodiester bond. Because of the extensive polar and hydrophobic interactions between 2’3’- cGAMP and STING binding sites, it has a higher affinity with STING than other CDNs (3’3’, 2’2 ‘, 3’2’ - cGAMP or c-di GMP), and is a ligand with higher affinity for STING (4, 36). STING is a small protein (~40 kDa), which contains a short N-terminal cytoplasmic segment, four transmembrane (TM) helices, and is located in the endoplasmic reticulum (ER) membrane, the cytoplasmic ligand binding domain (LBD), and the C-terminal tail (CTT) responsible for binding TBK1 (47). STING is a transmembrane receptor protein stimulator (STING) of interferon genees residing on the endoplasmic reticulum (ER) membrane (45). Under steady state conditions, STING binding ER forms a dimer, producing a V-shaped pocket ligand binding domain (LBD) that can bind to a cyclic dinucleotide, enabling cGAMP to bind STING (4). The combination of cGAMP and STING leads to its transfer from the endoplasmic reticulum to the Golgi apparatus, which is activated by palmitoylation on Cys88 and Cys91 (28, 36). TBK1 was recruited onto the conservative PLPLRT/SD motif at the C-terminal tail of STING (48), and IFIT3 was used as the linker connecting TBK1 and STING (36). Ligand binding and STING oligomerization seem to promote the trans phosphorylation of TBK1. Then active TBK1 phosphorylates the C-terminal tail (CTT) of STING (this occurs on the adjacent dimer of STING, not on the dimer bound by TBK1). STING CTT phosphorylation creates a docking site for IRF3, which is then phosphorylated by TBK1. IRF3 was then dimerized to obtain transcriptional activity (4). In Golgi apparatus, STING-TBK1 interaction finally activates interferon regulatory factor 3 (IRF3) and NF-κB that the main effectors of these two innate immunities (4, 28, 36). The combination of cGAMP and STING leads to the activation of different downstream effector functions, including autophagy and cell death (45).

In addition to its main catalytic function, cGAS also has a variety of non catalytic functions. cGAS can promote the perception of extracellular cyclic dinucleotides, making extracellular cyclic dinucleotides enter cells through endocytosis. These extracellular cyclic dinucleotides enhance the formation of the active dimer form of cGAS, indicating that they may be able to transfer equilibrium to cGAS dimer, thereby promoting the activation of cGAS (4, 28). The function of cGAS in human body is extensive, and the regulation of cGAS activity can play an important role in the treatment of diseases. Therefore, the study of cGAS agonists is hopeful to increase the success rate of treatment of related diseases.

## 3 Medical application of MnO_2_ nanomaterials

### 3.1 Introduction of MnO_2_ nanomaterials

MnO_2_ nanomaterials have been reported to show excellent physical, chemical and biological properties, such as high specific surface area, high adsorption, strong oxidation, high catalytic activity, broad spectrum light absorption range, strong fluorescence quenching, good degradability and biosafety, etc (49–51). There are two widely known mechanisms for MnO_2_ nanomaterials to excute their functions in biological systems: the redox reaction between MnO_2_ and glutathione (GSH) (52), and the catalytic reactions of MnO_2_ nanomaterials to decompose H_2_O_2_ into O_2_ under acidic conditions (53). These characteristics make MnO_2_ nanomaterials a kind of outstanding multi-functional biomedical materials that can be used in many biomedical fields, such assensors (54), contrast agents (55), biological tumor therapy (56) and drug delivery (57).

### 3.2 Preparation method of MnO_2_ nanomaterials

Due to the attractive properties, the preparation methods of MnO_2_ nanomaterials have been widely developed recently (As shown in **Table 1**). The template method is a typical method to prepare MnO_2_ materials with templates of different forms, and then etch the templates to obtain MnO_2_ products. For example, Fei et al. reported a method to prepare hierarchical hollow manganese dioxide nanosheets with intricate and well-controlled 3D morphologies by combining the Kirkendall effect with a sacrificial crystalline template (58). Particularly, discrete spherical and cubic hollow MnO_2_ nanostructures with controlled morphologies can be prepared by changing the morphologies of MnCO_3_ precursors, which can be simply obtained by adding the (NH_4_)_2_SO_4_ solution in the reaction system. Moreover, the thicknesses of the shells of hierarchical hollow nanostructures can be adjusted easily by the relative quantities of KMnO_4_ reacted followed by selective removal of MnCO_3_ crystal template. This template approach will find wide acceptance and use in the field of template-directed nanostructure synthesis of MnO_2_ nanomaterials.

**Table 1:** Typical preparation method of MnO2 nanomaterials.

Methods	Principle	Advantages	Disadvantages	Example
The template method	MnO_2_ nanomaterials were prepared with different forms of templates, and then the templates were etched to obtain manganese dioxide products.	1. The size, shape, structure, and properties of nanomaterials are precisely controlled by using templates as carriers. 2. Realizing the integration of synthesis and assembly of nanomaterials, and solving the dispersion and stability of nanomaterials.	1.Low reaction efficiency 2.Possibility of material damage when removing template	Fei et al. reported manganese dioxide nanosheets (58)
Hydrothermal method	Use high temperature and high pressure aqueous solution to dissolve those substances that are insoluble or difficult to dissolve under atmospheric conditions, or react to produce the dissolved products of the substances, and control the temperature difference of the solution in the high pressure gold to generate convection to form supersaturated state to produce long crystals.	1. high particle purity, good dispersion, good crystal shape and controllability, and low production cost. 2. The product does not need to be sintered to avoid grain growth and impurity mixing.	1.Strict temperature and pressure control 2.High equipment requirements	Guan et al.introduced a novel manganese dioxide-graphene nanosheets (GNSs) (59)
Redox Method	MnO_2_ is prepared by reduction of Mn^7+^(KMnO _4_) or oxidation of Mn^2+^(MnCl _2_) by Redox.	1. Simple operation, low cost, mild synthesis conditions and energy saving.	1. waste liquid pollution 2. Possibility of structural defects	Oaki et al. reported manganese oxide nanosheets (60)
Biomineralization Method	Bioorganic substances (such as serum albumin) will guide the nucleation of Mn^2+^ through control or/and influence under physical and chemical conditions, and then Mn^2+^ will spontaneously form MnO_2_ through growth or oxidation in alkaline solution.	1. The nucleation, growth, orientation and assembly of minerals are controlled at the molecular level. 2. The advantages of low cost, convenience and environmental friendliness 3.Preparation of multifunctional nano therapeutic agent.	1. Difficulty in integrating multifunctional organic materials	Fang et al. introduced albumin-MnO_2_ gated hollow mesoporous silica nanosystem (61)

Hydrothermal method is a simple and relatively environment-friendly method based on self-assembly for MnO_2_ nanomaterials preparation. High quality single crystal MnO_2_ nanomaterials can be easily synthesized by simple hydrothermal treatment. For example, Guan et al.introduced a facile one-step hydrothermal method to synthesize a novel manganese dioxide-graphene nanosheets (GNSs) (59). GNSs were fully mixed with KMnO_4_ in an acidic environment, and KMnO_4_ was then reduced to MnO_2_ by a hydrothermal process to allow the nucleation and growth of MnO_2_ on GNSs for biosensing application. Tehseen et al. Also introduced a hydrothermal method to prepare manganese oxide nanofibrous (MnO_2_ NFs) by mixing manganese acetate tetrahydrate and sodium hydroxide for hydrothermal reaction (62). Manganese acetate tetrahydrate is converted into manganese hydroxide (II) and then manganese (III) hydroxide upon the addition of alkali. And the hydrothermal treatment occurs under alkaline reaction conditions at elevated temperature, manganese (III) hydroxide converts to manganese (IV) oxide (blackish brown) that builds the nuclei for the growth of nanomaterials. These hydrothermal methods can synthesize MnO_2_ nanomaterials with facile, one-pot, and cost-effective approach without using any template or structure directing compound.

Oxidation and reduction methods are also widely used for MnO_2_ nanomaterials preparation, which mainly include Mn^2+^ (manganese chloride) oxidation method and Mn^7+^ (potassium permanganate) reduction method. Manganese dioxide can be synthesized by oxidizing Mn^2+^ or reducing potassium permanganate with reducing agents and oxidants, to form manganese dioxide nanostructures. Oaki et al. reported the method for one-pot synthesis of manganese oxide nanosheets in an aqueous solution by mixing solutions containing Mn^2+^/EDTA and NaOH (60). Chelation to the divalent manganese ion and interaction with the resultant oxide involved to form birnessite nanosheets in aqueous solution at room temperature, which finally led to the formation of manganese oxide nanosheets. Yang et al. proposed a method for preparing MnO_2_ nanoparticles by reducing KMnO_4_ with excessive Na_2_S_2_O_3_ and the GOx and BSA-Chlorine e6 (BSA-Ce6) were employed onto the surface of MnO_2_ nanoparticles (NPs) (63). These oxidation reduction methods are widely used as a convenient, simple and effective method to prepare manganese dioxide materials without any specific requirements of hydrothermal treatment and special equipment.

Biomineralization is a technology that combines biological macromolecules with inorganic materials to prepare inorganic minerals by adjusting biological macromolecules for MnO_2_ nanomaterials preparation. Bioorganic substances (such as serum albumin) can convert ions into solid minerals under physical and chemical conditions (61), which is also partially based on the oxidation and reduction methods. During the preparation process of MnO_2_, biological organic substances guide the nucleation of Mn^2+^ to spontaneously form MnO_2_ nanostructures through growth or oxidation in alkaline solution (64). The biomineralization method provides the feasibility to synthesize multifunctional and biocompatible MnO_2_ nanomaterials as potential therapeutic agents (65). These biomineralization methods have demonstrated the advantages of cost-effectiveness, convenience and environmental friendliness, which thus can be widely applied for dioxide nanomaterial preparation.

### 3.3 Basic biological activity of MnO_2_ nanomaterials

The excellent physical, chemical and biological properties of MnO_2_ nanomaterials make it an excellent multifunctional biomaterial and can be used in many biomedical fields (66). MnO_2_ nanomaterials have been widely developed as drug carriers to enhance drug efficiency. The drugs used to treat tumors will reduce the effect of chemotherapy because of the drug resistance factors and hypoxia conditions in the tumor microenvironment, such as overexpression and activation of hypoxia inducible factors (eg, HIF-1a) and multidrug resistance (MDR) (eg, multiple p53 and P-glycoprotein [P-gp]). Manganese dioxide can improve the tumor microenvironment and make the drug loaded complex show better efficacy than single drug. Amini et al. proposed a strategy that preparing multifunctionality of polymer-lipid encapsulated MnO_2_ nanomaterials (PLMD NPs) as drug carrier to enhance the efficacy of doxorubicin (56). These MnO_2_ nanomaterials can enhance the penetration of doxorubicin to tumors for enhanced chemotherapy efficiency. In addition, manganese dioxide can reduce hydrogen peroxide in the microenvironment, reduce oxidative stress, and protect biological enzyme drugs. Manganese dioxide materials help biological enzyme drugs overcome the shortcomings of low stability, high cost and storage difficulties of natural enzymes, and improve their potential applications in the biomedical field. E. Marin et al. proposed a method to prepare an antioxidant microreactor by encapsulating manganese dioxide nanoparticles into layered polymer capsules (67). They used manganese dioxide nanoparticles to replace the encapsulation of traditional antioxidant enzymes, providing a more solid and stable inorganic substitute, which is a new strategy for preparing antioxidant polymer microreactors. Li et al.introduced chitosan-modified hollow MnO_2_ nanomaterials as a resveratrol loading system, which indicated that MnO_2_ nanomaterials could also serve as a potential central nervous system drug delivery system for the treatment of spinal cord injury (68). Wang et al. show that a leukemia cell membrane (LCM)-camouflaged hollow MnO_2_ nanocarrier (HM) with encapsulated doxorubicin (DOX) (denoted LHMD) could bind specifically to AML cells with a homologous targeting effect (69). LHMD rationally deliver chemotherapeutic agents and to trigger Mn^2+^ mediated STING pathway activation for potent immune and chemotherapy against AML cells.

MnO_2_ nanomaterials can release Mn^2+^ in the body, which therefore makes MnO_2_ nanomaterials possess the biological functions of Mn^2+^. Mn^2+^ can be used as an effective T1 weighted MRI contrast agent because of its five unpaired 3d electrons (70).Zhang et al. constructed an intelligent nanoplatform based on poly (N-vinylcaprolactam) (PVCL) nanogels (NGs) co-loaded with gold (Au) and manganese dioxide (MnO_2_) nanoparticles (NPs) for dual-mode computed tomography (CT)/magnetic resonance (MR) imaging (71). MnO_2_ nanomaterials can release Mn^2+^ more efficiently under tumor microenvironment conditions, which therefore provided an enhanced signal for MRI imaging and can be used for imaging-guided therapy. Xiao et al. also evaluated the MRI imaging performance of MnO_2_ nanomaterials under simulated tumor microenvironment conditions, and Found that the relaxation rate under tumor microenvironment conditions was 16 times higher than that under non tumor microenvironment conditions (70). These experiments show that MnO_2_ nanomaterials can be used as a potential T1 contrast agent for tumor specific MRI imaging or imaging-guided therapy method development.

Tumor microenvironment (TME) is usually characterized by low pH value, high glutathione (GSH) concentration, excessive production of hydrogen peroxide (H_2_O_2_) and severe hypoxia (72). These characteristics can provide a favorable internal environment for the generation and survival of tumor cells, and are closely related to tumor progression, metastasis and drug resistance. Manganese dioxide (MnO_2_) is widely used in nanocomposites for MTE regulation due to their pH responsiveness and excellent catalytic activity. MnO_2_ nanomaterials can catalyze excessive H_2_O_2_ in TME to generate oxygen to alleviate tumor hypoxia (73), and exhaust GSH excessively to reduce the antioxidant capacity of TME (74). In the presence of glucose oxidase (GOx), manganese dioxide can also catalyze the conversion of glucose into reactive oxygen species (ROS) through a cascade Fenton like reaction for hunger treatment of tumors (11). Based on these properties, MnO_2_ nanomaterials can significantly increase the generation of oxygen and ROS, which can relieve the hypoxia of TME and induce ROS associated damage of tumor cells, synergistically enhancing the efficiency of chemotherapy and photodynamic therapy (74).

Taking the above advantages, MnO_2_ nanomaterials can also act as a kind of novel immune immunomodulators by regulating different immune cells.

The levels of immunosuppressants are significantly increased under the condition of tumor hypoxia, such as adenosine, lactic acid and other substances that show inhibition for the activation and killing function of macrophages and cytotoxic immune cells (75–77). Murphy et al., designed a kind of PLGA-encapsulated MnO_2_ nanomaterials to enhance the cytotoxicity of NK cells against cancer cells by improving hypoxia and reducing immunosuppressants (78). Liang et al. also found that Gold Nanocages @ Manganese Dioxide (AuNC@MnO_2_) nanosystem could reverse the immunosuppressed TME by relieving tumor hypoxia through *in situ* oxygenation, and further enhance the recruitment of CD8+ T cells to tumor tissue for enhanced anti-tumor efficiency (79). These effects were found to be associated with the ability of AuNC@MnO_2_ to induce immunogenic cell death (ICD) to release damage related molecular patterns (DAMPs), and induce DC maturation and effector cell activation. In addition, MnO_2_ nanoparticles were also proved to act as a minimalist multimode vaccine adjuvant/delivery system to regulate antigen presenting cells for tumor immunotherapy (80). These results collectively suggested the attractive uses of MnO_2_ nanomaterials for potential immunotherapy.

## 4 The significance of MnO_2_ for cGAS-STING pathway

### 4.1 How Mn^2+^ involved in cGAS-STING pathway

cGAS-STING can regulate tumorigenesis, participate in autoimmunity and is also essential for the host defense against different kinds of pathogens (81). Thus, the active regulation of cGAS-STING signaling events is expected to develop novel therapeutics against tumor and infections. Mn^2+^ is the trace in cytoplasm, mitochondria and Golgi apparatus, and has a highly synergistic relationship with dsDNA ligands as a metal cofactor (82, 83). During virus infection, Mn^2+^ is released from membrane wrapped organelles and then accumulated into cytosol, which can further directly binds to cGAS in the cytosol, promoting the binding of cGAS and dsDNA to activate cGAS (84). After being activated, cGAS cyclizes ATP and GTP into cGAMP (the second messenger), and activates the innate immune response mediated by type I interferon (IFN-I) through interferon gene stimulator (STING). Thus, Mn^2+^ plays an important role in host defense by activating cGAS (82), which shows strong potential for the development of novel therapeutics and vaccines.

### 4.2 How MnO_2_ nanomaterials transform into Mn^2+^ in cells

**Figure 2:**
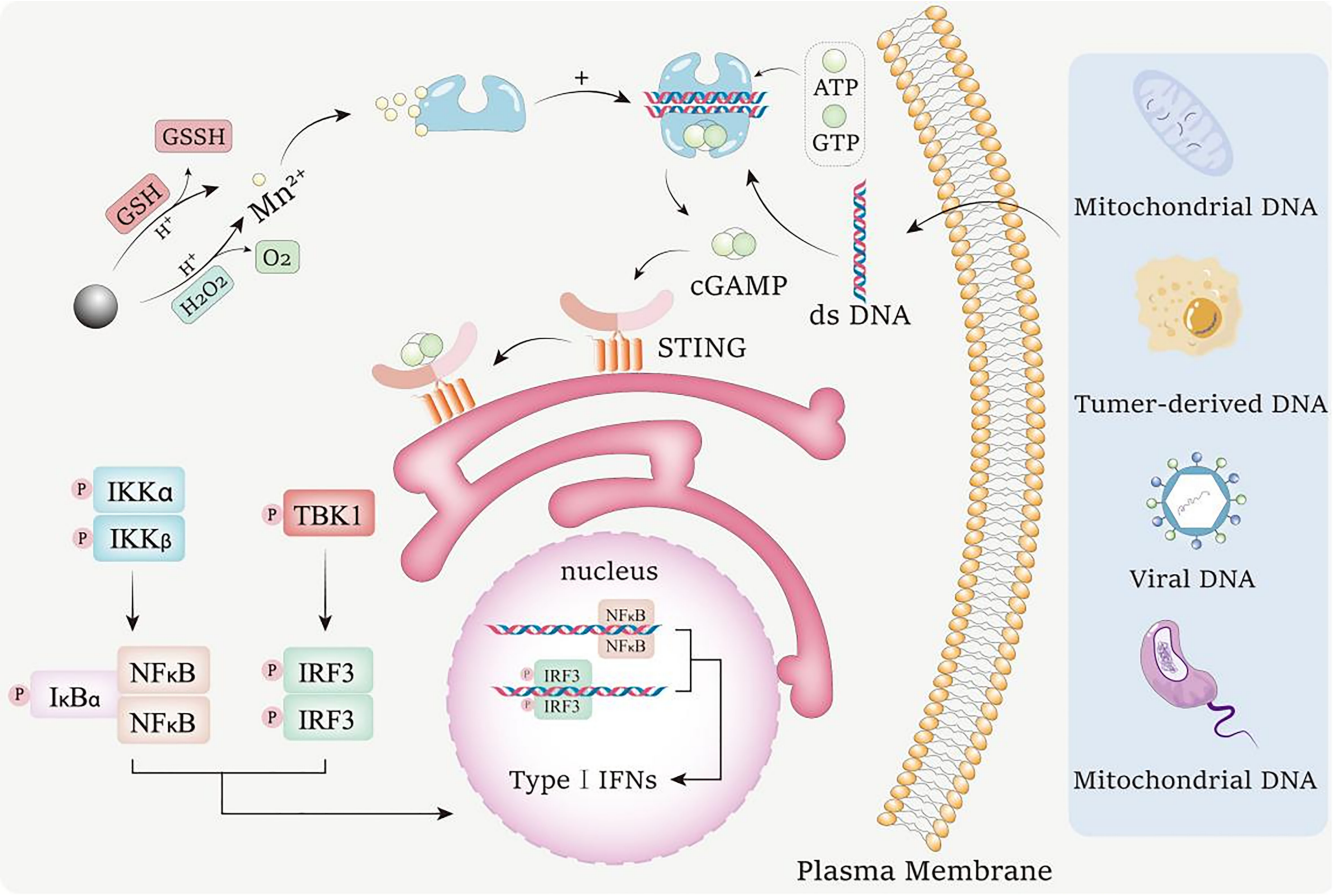
Mechanism for the conversion of MnO_2_ nanomaterials into Mn^2+^ to activate cGAS-STING pathway. MnO_2_ nanomaterials can undergo redox reactions with GSH and H_2_O_2_ in acidic tumor environments to consume excessive GSH and H_2_O_2_ in tumor environments. In response, Mn^2+^ is released, and the generated O_2_ can improve the tumor hypoxia state. Mn^2+^ enhances the ability of cGAS to recognize dsDNA from bacteria, tumor cells, viruses and mitochondria in the environment, and promotes the production of 2’,3’ - cyclic GMP AMP (2’,3’ - cGAMP). cGAMP binds to interferon gene stimulator (STING), resulting in tank binding kinase 1 (TBK1) dependent phosphorylation (P) of interferon regulator 3 (IRF3). The active IRF3 dimer transfers to the nucleus and activates the transcription of type I interferon gene. STING can also activate IκB kinase (IKK), phosphorylated NF-κB Inhibitory Protein IκB α, Lead to polyubiquitination and degradation, and then NF-κB can enter the nucleus and start the transcription of immune stimulating genes.

MnO_2_ nanomaterials execute lots of their functions in the body in the form of Mn^2+^. As shown in **Figure 2**, when MnO_2_ nanomaterials enter into the acidic tumor microenvironment, they can be reduced to Mn^2+^ by the excessive H_2_O_2_ and GSH in the environment (85, 86). By using these two pathways, MnO_2_ nanomaterials can be transformed into Mn^2+^ to achieve multiple biological activities.

The release of Mn^2+^ from MnO_2_ nanomaterials is a process of reduction. Firstly, MnO_2_ nanomaterials can act as an oxidant in the reaction with H_2_O_2_ (87). MnO_2_ oxidizes H_2_O_2_ to O_2_ and generates MnOOH, which is an intermediate in the process of Mn^2+^ generation. Then, MnOOH is also used as an oxidant to oxidize H_2_O_2_ into H_2_O and O_2_ under an acidic environment, while MnOOH itself is reduced into Mn^2+^. When H_2_O_2_ is insufficient in the reaction system, MnOOH can undergo a disproportionation reaction under acidic conditions to generate tetravalent MnO_2_ and Mn^2+^. The reaction formulas during the reactions between MnO_2_ and H_2_O_2_ are as shown as following:

$2MnO_{2}+H_{2}O_{2}\to 2MnOOH+O_{2}$

$2MnOOH+4H^{+}+H_{2}O_{2}\to 2Mn^{2+}+4H_{2}O+O_{2}$


$$2MnOOH+2H^{+}\to M^{n}O_{2}+Mn^{2+}+2H_{2}O$$

Moreover, MnO_2_ nanomaterials can also interact with GSH to release Mn^2+^. It has been widely reported that there is excessive GSH in the tumor microenvironment, which provides sufficient reductant for the reduction of MnO_2_ (88). Under acidic conditions, GSH is oxidized to glutathione disulfide (GSSG) through mercaptan disulfide exchange, while MnO_2_ is reduced to Mn^2+^ (89). The reaction formula during the reaction between MnO_2_ and GSH are as shown as following:


$$MnO_{2}+2GSH+2H^{+}\to Mn^{2+}+GSSG+2H_{2}O$$

The tumor microenvironment provides acidic conditions for the above series of reactions. These Mn^2+^ released from MnO_2_ can participate in the subsequent immune responses for tumor inhibition, and the hypoxia conditions in the tumor microenvironment are also improved while the GSH level is downregulated, which makes the tumor more sensitive to ROS-mediated inhibition.

### 4.3 Mechanisms of Mn^2+^ activation of cGAS-STING pathway

Mn^2+^ can enhance the nucleotide transferase (NTase) activity of cGAS to substrate, especially the homologous substrate ATP/GTP. The non-homologous substrates ATP/ATP and GTP/GTP can also be cyclized when cGAS binds to short dsDNA, but the products can’t activate STING. When cGAS binds to long dsDNA, the homologous substrate ATP/GTP is cyclized into cGAMP to activate STING and induce subsequent signal transmission. This method, which depends on the length of cytoplasmic dsDNA, is the key to measuring stress levels by cGAS. Long dsDNA (≥ 50 base pairs (bps)) always indicates disease status, such as the presence of viral genome or mislocation of mitochondrial dsDNA, while shorter segments always indicate slight DNA damage or degraded viral genome (82). Mn^2+^ can combine with cGAS to improve the sensitivity of cGAS to dsDNA, so that cGAS can recognize and combine with dsDNA in low dsDNA concentration environment, and enhance the binding rate of cGAS and dsDNA (90, 91). This ability reduces the requirement of dsDNA concentration for the activation of cGAS, and improves the activation efficiency of cGAS. Manganese also has positive effects on the length dependence of dsDNA to reduce the length dependence of cGAS activation on dsDNA by directly enhancing the catalytic activity. This reduces the effect of dsDNA length on the cGAS activation intensity (82)]. In disease environments that require high levels of cGAS activation, such as tumor environments, this function of Mn^2+^ has great potential in the immunotherapy of diseases.

More interestingly, Mn^2+^ can activate cGAS alone in the absence of dsDNA, and the maximum activation activity in the absence of dsDNA in the system is equivalent to that induced by dsDNA. Thus, Mn^2+^ can directly combine with cGAS to activate cGAS signalings in a DNA independent manner with different mechanisms (83). When dsDNA binds to cGAS, the dimerization and oligomerization of cGAS are critical to the activation of cGAS. However, the cGAS activated by Mn^2+^ is independent of the typical dimerization triggered by dsDNA. Intact DNA-cGAS interactions, mediated by both the N-terminal R/K-rich region and the DNA-binding sites at the C-terminal male abnormal 21 (Mab21) domain, are critical for cGAS activation in cells. However, Mn^2+^ can also activate cGAS in mutants lacking both of these terminals. Structural analysis shows that the conformational changes of cGAS are similar to those of dsDNA induced cGAS activation, but shows unique η1 Helix to expand the catalytic pocket, allowing substrate entry and cGAMP synthesis (83). This means that Mn^2+^- cGAS has experienced a conformational change similar to that of dsDNA -cGAS, but shows differences in the unique η1 helix formed in Mn^2+^ activated cGAS. This structure leads to a significant widening of the entrance of the catalytic pocket, making the active site close to the substrate. However, the tightness of cGAS binding to Mn^2+^ is 85 times lower than that in the presence of dsDNA (82).

The way that Mn^2+^ activates cGAS to synthesize cGAMP is also different from that of dsDNA. In the presence of Mn^2+^, cGAS synthesizes cGAMP by a unique mechanism. In the process of cGAMP synthesis, there will be the formation of intermediate products, linear dinucleotide pppGpG. In the cGAS-Mn^2+^- pppGpG ternary complex, compared with the dsDNA activated cGAS, 5’pppG (2’5’) pG binds to cGAS in the opposite direction. Two guanine residues are linked by a 2’5’phosphate bond with the first and the second guanine adapting anti and syn conformation. In the structure of cGAS-Mn^2+^- pppGpG, the two cations (Mn^2+^) are only coordinated by the triphosphate group of the bound dinucleotide, independent of the catalytic triad residues (Asp211, Glu213 and Glu307) required by mcGAS-dsDNA pppGpG. Unlike the previously reported structures of DNA-activated cGAS, in cGAS-Mn^2+^ 5’pG(2’5’)pA and cGAS-Mn^2+^- c[G(2’5’)pA(3’5’)p], the adenine residues of both dinucleotides stacked with Tyr421, whereas the guanines located at the opposite side, which implies that the linear intermediate would not have to flip over within the catalytic pocket before being cyclized to accelerate the overall catalytic activity of cGAS. And by binding with cGAS to synthesize cGAMP, Mn^2+^ can enhance the surface STING function of the endoplasmic reticulum, then further enhance IRF3 phosphorylation, leading to the amplification of STING signal cascade and the production of type I IFNs (92).

### 4.4 MnO_2_ nanoparticles based therapeutic strategies by manipulating cGAS-STING pathway

Tumor remains a substantial challenge to human health with millions of deaths every year worldwide. Stimulator of interferon genes (STING) signal activation is a significant component to enhance innate immunity, which has been used to realize broad-spectrum immunotherapy. Thus, cGAS-STING pathway has been regarded as one of the most promising targets for tumor therapy, but how to develop an effective strategy to control cGAS-STING pathway for tumor therapy is still a big challenge.

Different forms of manganese dioxide nanocomposites have shown excellent performance in the three parts of cancer treatment, including chemotherapy, phototherapy, and collaborative treatment (93). In the field of cancer treatment, manganese dioxide nanomaterials show superior advantages and unprecedented performance. Mn^2+^ containing nanomaterials is expected to directly activate cGAS-STING signaling. For example, Wang et al. described a bovine serum albumin (BSA)/ferritin-based nanoagonist incorporating Mn^2+^, which could activate cGAS-STING signaling in dendritic cells (DCs) to elicit robust adaptive anti-tumor immunity. Mn^2+^ could enhance the sensitivity of cGAS to dsDNA and augment STING signaling, which could further enhance the tumor-specific T cell-mediated immune response against poorly immunogenic solid tumors *in vivo*, offering a robust approach for immunotherapy in the clinics. However, Mn^2+^ is not stable and very easily to be oxidized, which requires more stable Mn containing nanoparticles that can steadily provide Mn^2+^ for effective activation of cGAS-STING pathway.

Taking the advantages of high stability and biocompability, MnO_2_ nanoparticles have been demonstrated to show strong anti-tumor activities by releasing manganese ions in the tumor environment to enhance the activation of cGAS-STING pathway. This promising cGAS-STING regulating function is widely used for the development of various therapeutic anti-tumor strategies based on MnO_2_ nanomaterials. For example, Song et al. built a MnO_2_ contained M@P@HA nanoparticles as novel cGAS-STING amplifiers to enhance the innate immunotherapy against tumor (94). These M@P@HA nanoparticles, with photodynamic therapy (PDT) reagent protoporphyrin (PpIX) loading, could generate ROS-mediated disruption of cellular redox homeostasis and cytoplasm leakage of damaged mitochondrial double-stranded (ds) DNA under laser irradiation. Moreover, the M@P@HA nanoparticles can be decomposed for MnO_2_ exposure, which could interact with the intracellular GSH to release Mn^2+^. These released Mn^2+^ could significantly activate cGAS and increase the activity of the related proteins of cGAS-STING signaling to further amplify the anti-tumor immunity of tumor-associated macrophages (TAM). They also found that, by amplifying the cGAS-STING signaling of tumor tissue and activating innate immunity, this nanosystem can also further activate CD8+ T cell infiltration even in a tumor with low immunogenicity for synergistic tumor therapy.

MnO_2_ nanomaterials have been widely used to play a role as biocompatible nano carriers, which can be combined with their cGAS-STING activation for more effective tumor treatment. Lu et al. reported an HMnMPH nanoplatform consisting of hollow manganese dioxide (HMn) loaded with STING agonist (MSA-2), CRISPR-Cas9/sg-PD-L1 plasmid and hyaluronic acid (HA) (95). In tumor microenvironment, HMnPMH can be degraded to release Mn^2+^ and STING agonists, which can promote the release of type I interferon and proinflammatory factors by continuous activation of cGAS-STING pathway. CRISPR-Cas9 plasmid released by the nanosystem can also relieve PD-L1 immune checkpoint and reactivate immunosuppressive T cells, which can trigger long-term immunotherapy by combining with the cGAS-STING pathway mediated innate immunity activation. Du et al. also prepared a pH/glutamate responsive drug loading hollow manganese dioxide (H-MnO_2_) - based chlorine6 (Ce6) - modified DNAzyme thermal nanosystem to combine gene therapy and immunotherapy (96). The biodegradation of H-MnO_2_ releases DNase as PD-L1 mRNA targeting reagents to activate gene therapy with the assistance of DNase cofactors. The released Mn^2+^ is also involved in tumor immunotherapy through immune activation of the cGAS-STING pathway. And ROS produced by photosensitizers Ce6 and GA could further lead to immunogenic cell Death for more effective tumor inhibition. Yang et al. created an intelligent biodegradable hollow manganese dioxide (H-MnO_2_) nano-platform (97). H-MnO_2_ with hollow structure can be loaded with anti-tumor drugs, and can be used for specific imaging and on-demand drug release in tumor microenvironment (TME), and can also be used to regulate hypoxia TME to enhance cancer treatment.

Similarly, Du et al. reported a nanoplatform naming CA-1@HMnO_2_/HA-P-mAb for potential cancer chemoimmunotherapy (98). MAb, an antibody against Neuropilin-1 (Nrp-1) on regulatory T cells (Tregs), endows the nanosystem Tregs targeting effects to avoid the transmission of inhibitory signals to T cells. MnO_2_ inside the nanosystem can deplete the overexpressed GSH in tumor cells to produce Mn^2+^, which can activate the cGAS-STING pathway in 4T1 cells and up regulate the expression of IFN to promote dendritic cell maturation and tumor specific antigen presentation. Similar to curcumin (Cur), CA-1 has the characteristics of inducing tumor death through chemotherapy to enhance tumor immunogenicity, and effectively recruit CD4+T helper cells (Th) and CD8+ cytotoxic T lymphocytes (CTL) for the enhancement of anti-tumor immune responses.

MnO_2_ nanomaterials can also regulate the anoxic tumor microenvironment, which can be combined with the activation of cGAS-STING pathway for more effective anti-tumor treatment. Liu et al. reported the biomineralized manganese oxide nanoparticles (bio-MnO_2_ nanoparticles) prepared by mild enzymatic reaction (99). Bio-MnO_2_ nanoparticles can react with endogenous H_2_O_2_ to produce O_2_ and catalyze ROS production in tumors, thereby improving the radiosensitivity of NSCLC cells. At the same time, the released Mn^2+^ activates the activity of cGAS-STING pathway, promotes the death of immunogenic cells, and increases the infiltration of cytotoxic T cells. These results indicate that MnO_2_ nanoparticles also play an important role in RT induced anti-tumor immune response in non-small cell lung cancer (NSCLC). Zhao et al. prepared a MnO_2_ coated gold nanorods (GNRs) with silicon dioxide (SiO_2_) covering, and further camouflaged with myeloid suppressor cells (MDSCs) membrane to form GNRs@SiO_2_ @ MnO_2_@MDSCs (GSMM) (18). MnO_2_ can release Mn^2+^ in acidic micro tumor environment, which catalyzes H_2_O_2_ into O_2_ for anoxic tumor microenvironment remission and ·OH for CKT (chemical kinetics therapy). Moreover, Mn^2+^ can activate cGAS-STING to induce the secretion of type I interferon, proinflammatory cytokines and chemokines. At the same time, the NIR-II window photothermal imaging and photoacoustic imaging of GSMM are realized through the local surface plasmon resonance of MnO_2_ coating and GNRs in the near-infrared II window. PTT and CDT mediated tumor cell immunogenic cell death can further enhance anti-tumor immunity.

Cancer vaccines are viewed as one of the most promising immunotherapy strategies to eradicate malignant tumors by eliciting tumor-specific immune responses. However, the development of cancer vaccines is significantly restricted by the low immunogenicity of antigens and invalidation of adjuvants. To develop a novel cancer vaccine, Tang et al. designed a TME responsive MnO_2_ melittin nanoparticles (M-M NPs) to consume GSH *via* Fenton-like reaction (100). The released Mn^2+^ could further activate cGAS-STING signaling and promote the maturation of antigen-presenting cells to elicit systemic anti-tumor immune responses, such as the augmentation of tumor-specific T cells and pro-inflammatory cytokines/chemokines production. More importantly, M-M NPs could also promote the MHC-I cross-dressing by DCs to prime tumor-specific CD8+ T cells. These results proposed a strategy to enhance the cancer vaccine efficiency which showed great therapeutic effect on tumor immunotherapy.

MnO_2_ nanomaterials can also act as immune adjuvants and synergists for tumor treatment. Li et al. reported MnO_2_@MPDA-PEG nanoparticles, through the incorporation of MnO_2_ into polyethylene glycolate mesoporous polydopamine nanoparticles (MPDA-PEG nanoparticles) as a kind of mild photothermal effect assisted nanosystem (101). In colorectal tumors, nano drugs can release Mn^2+^ in the tumor microenvironment, which can be used as immune adjuvants to promote DC maturation and T cell activation. At the same time, it can improve the tumor microenvironment and make the tumor change from “cold” to “hot”, thus promoting the immunotherapy of aPD-L1. Mn^2+^, a magnetic resonance imaging (MRI) contrast agent for tumor imaging, can also induce BMDC maturation under mild photothermal treatment. This MnO_2_@MPDA-PEG nanosystem thus could be a promising dual-imaging theragnostic nanodrug to potentiate the systemic antitumor immunity. Song et al. used model antigen ovalbumin (OVA) to construct tumor vaccine OVA/MnO_2_ to test the mechanism and efficacy of MnO_2_ based tumor vaccine adjuvant/delivery system (80). As an advanced adjuvant pool, MnO_2_ nanoparticles can continuously release Mn^2+^ and enhance the immune response through the cGAS-STING pathway in DCs. These MnO_2_ nanoparticles can also be used as an ideal delivery system to deliver antigens to the cytoplasm of DCs to induce cellular immune response, indicating their promising roles as minimalist multi-mode vaccine adjuvant/delivery system. Liang et al. constructed TDNeMnO_2_ complex by combing tetrahedral nanostructured DNA (TDNs) with MnO_2_ nanosheet to activate macrophages (102). TDNs and Mn^2+^ have synergistic effects in triggering cGAS-STING activation, production of Ifnb/Isgs, M1 polarization and antigen presentation, providing a new anti-tumor immunotherapy strategy based on STING mediated TAM reprogramming. Zheng et al. constructed a Mn-enriched photonic nanodrug (MnPB MnOx), which is made by integrating MnOx onto the surface of Mn-doped PB nanoparticles. All components of MnPB MnOx have biocompatibility and biodegradability, and sufficient Mn ensure efficient activation of cGAS-STING, thus activating innate immunity. MnPB enhances tumor hyperthermia under near-infrared radiation, and cooperates with MnOx which can catalyze the production of reactive oxygen species, further promoting the stimulation of immune response. In the system of bilateral in-situ 4T1 tumor-bearing mice, the innate and adaptive immunity has been successfully activated, and the local treatment can produce the function of inhibiting the systemic response of distant tumors has been confirmed (103).

## 5 Conclusion and perspectives

MnO_2_ nanomaterials have indicated strong potential to activate the cGAS-STING pathway for enhanced innate immunological responses. Here we discussed the excellent biological properties and preparation methods of MnO_2_ nanoparticles, as well as the importance of cGAS-STING signaling pathway in diseases and their specific mechanisms. Furthermore, we emphatically discussed how MnO_2_ nanomaterials activate cGAS by converting into Mn^2+^, as well as the application of MnO_2_ nanomaterials for disease treatment of diseases by mediating cGAS-STING pathway.

cGAS-STING signal pathway, a natural immune pathway that has attracted increasing attentions in recent decade, plays an important role in cellular immune responses. The ability of cGAS to recognize and combine with DNA can activate cGAS to catalyze the formation of cyclic dinucleotides (cGAMP) for the activation of the downstream cascade cGAS-STING signaling pathway, which can promote the transcription of type I interferon (IFN) (104). Type I IFNs can further promote DC maturation, antigen presentation, T cell activation, infiltration and other aspects, thus promoting adaptive immunity.

Mn^2+^ has been proved to show cGAS-STING activation activities as a novel cGAS agonist (81). Although Mn^2+^ based nanomaterials can be directly used to activate cGAS-STING signaling, their biomedical uses are dramatically restricted by their low stability and biocompatibility. MnO_2_ nanomaterials are a kind of manganes-based multifunctional biomedical materials with excellent physical, chemical and biological properties. Interestingly, as shown in **Figure 2**, MnO_2_ nanomaterials can be converted into Mn^2+^ by the reactions with intracellular H_2_O_2_ or GSH (105), which thus provide the possibilities for cGAS-STING signaling regulations using MnO_2_ nanomaterials.

The reserve of manganese is huge, the preparation of manganese based materials is simple, the cost is low, and its transportation and storage are very convenient. As an activator of the cGAS-STING pathway, Mn^2+^ can enhance the sensitivity of cGAS to detect DNA and promote the synthesis of cGAMP with a ten-thousand-fold efficiency, and enhance the binding ability of STING and cGAMP hundreds of times. Moreover, Mn^2+^ can directly activate cGAS without relying on dsDNA. Manganese-based materials can be used as drug carriers and improve the tumor environment, and enhance the efficacy of chemotherapy, phototherapy, and cooperative therapy on tumors.

**Figure 3:**
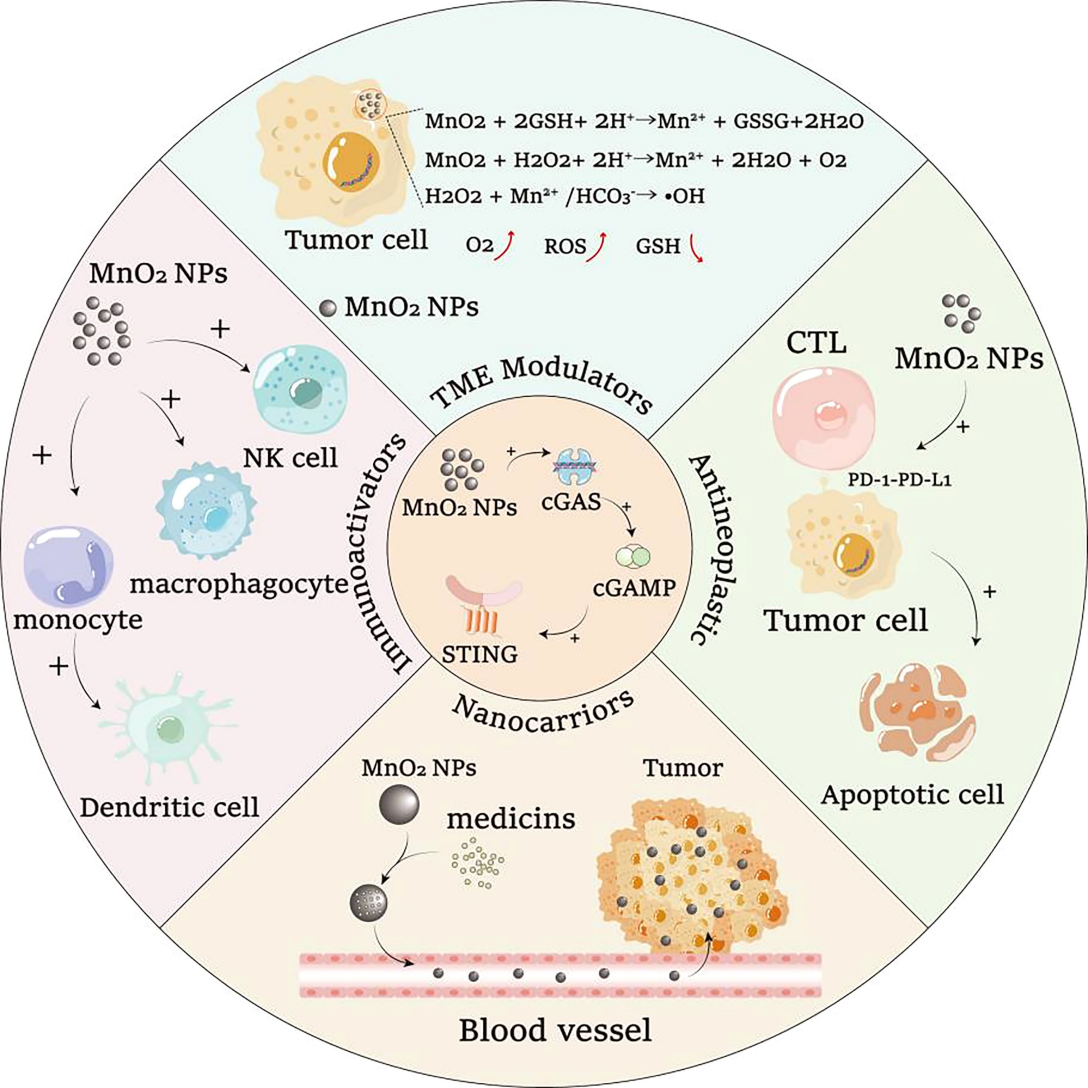
The ability of MnO_2_ nanomaterials to activate cGAS-STING signaling pathway can cooperate with other biological functions of MnO_2_ nanomaterials for potential synergistic treatment of diseases.

Based on the ability to activate cGAS and promote cGAS-STING signaling pathway, MnO_2_ nanomaterials can be used for the development of chemotherapeutic synergists, immune adjuvants and vaccines. To enhance drug efficiency, MnO_2_ nanomaterials can act as drug carrier for targeted drug delivery, which could also result in the reduced side effects of drugs (106). Moreover, as shown in **Figure 3**, the cGAS-STING pathway activating functions of MnO_2_ nanomaterials could allow the use of MnO_2_ nanomaterials as synergists to further enhance the efficacy of drug treatment. Additionally, MnO_2_ nanomaterials can also serve as immune adjuvants to enhance the innate and adaptive immunity, which could further improve the development of more effective vaccines. And for its cargo loading roles, MnO_2_ nanomaterials can also be loaded with the active cargo of vaccine for targeted delivery of the vaccines, which therefore can enhance the efficiency of vaccines more effectively by combining with their adjuvant roles.

Up to now, most of the studies of MnO_2_ nanomaterials are focused on their anti-tumor application, with few of studies for anti-infectious treatments. In fact, as one of the most important innate immunological regulation pathways, cGAS-STING signaling plays critical roles in virus and bacteria infection by regulating both innate and adaptive immunity. Mn^2+^ has also been proved to show anti-viral activities by regulating cGAS-STING signalings (84). And due to the potent cGAS-STING activation ability, we believe that MnO_2_ nanomaterials could be served as novel cGAS agonist for anti-infectious therapeutic and vaccine development.

Moreover, although MnO_2_ nanomaterials have showed attractive potentials for the development of novel treatments and vaccines by regulating cGAS-STING pathway, there are still some critical issues, such as their cytotoxicity, immunogenicity and pharmacokinetic and metabolism, are still needed to be further investigated. Up to now, the systemic safety of MnO_2_ nanomaterials *in vivo* within a long duration, which is critical for the biological and medical application of MnO_2_ nanomaterials, remained to be further evaluated. Although the mechanisms of MnO_2_ nanomaterials to activate cGAS-STING pathway through the forms of Mn^2+^ has been depicted by different studies, the wide-range immunogenicity of MnO_2_ nanomaterials have not been well demonstrated. At present, the research of MnO_2_ nanomaterials is in the early stage, and stability is the key factor that should be considered in biological experiments. Whether the toxicity and degradation of MnO_2_ materials will be affected by their different forms and sizes, as well as their combination with different drugs and materials. To ensure the safety of further potential clinical applications, a detailed biological and biological safety assessment of MnO_2_ and its nanocomposites is required. Although there are still some unsolved problems, innovative applications based on MnO2 and its nanocomposites still have great potential. Moreover, the *in vivo* pharmacokinetic and metabolism of MnO_2_ nanomaterials, which are very important for the biosafety uses of MnO_2_ nanomaterials, are also needed to be further investigated in different animal models. The systemic studies of these issues would benefit our understandings of the biosafety for MnO_2_ nanomaterials and facilitate the development of novel MnO_2_ nanomaterials for clinical uses.

At present, STING agonists of CDNs also exist. When CDNs STING agonists are injected intravenously, they will be consumed at non-tumor sites and play a role in making STING abnormally excited in normal tissues, which may lead to inflammation or autoimmune diseases (107). Therefore, intratumoral injection is required, but this limits its immune function. MnO_2_ and its composite nano-materials can target tumors in the form of responding to the tumor microenvironment and loading and modifying targeted biological agents, and play a role in tumors. These characteristics enable MnO_2_ and its composite nano-materials to be injected intravenously and give full play to the immune function. At present, various reports on the activation of STING by MnO_2_ and its composite nanomaterials have shown good results. The multi-form and loading function of MnO_2_ and its composite nano-materials is a huge breakthrough in innovation, and there is a huge potential to replace traditional agonists and apply them in clinical practice.

In conclusion, MnO_2_ nanomaterials have demonstrated strong potentials as novel cGAS agonists to activate cGAS-STING pathway. Although lots of issues need to be further investigated and clarified, we believe that the promising activity of MnO_2_ nanomaterials for cGAS-STING regulation would benefit the development of novel treatments and vaccines against some important diseases, such as tumor and infection.

## Author contributions

TZ, CH, WZ and YR drafted this manuscript and share first authorship. YM, DC, YHH, SF, WL, YFH, KL, and HL helped to revise the manuscript. J-FX, JP and XG helped to revise the manuscript and were responsible for leading this work. All authors contributed to the article and approved the submitted version.
